# Explaining Neighbourhood Variations in the Incidence of Dengue Fever in Jeddah City, Saudi Arabia

**DOI:** 10.3390/ijerph182413220

**Published:** 2021-12-15

**Authors:** Ibrahim Alkhaldy, Ross Barnett

**Affiliations:** 1 Department of Administrative and Human Research, Umm Al-Qura University, Makkah 21955, Saudi Arabia; 2 School of Earth and Environment, University of Canterbury, Christchurch 8140, New Zealand; ross.barnett@canterbury.ac.nz

**Keywords:** dengue fever, Jeddah City, neighbourhood socioeconomic status, pathways

## Abstract

The rapid growth and development of cities is a contributing factor to the rise and persistence of dengue fever (DF) in many areas around the world. Many studies have examined how neighbourhood environmental conditions contribute to dengue fever and its spread, but have not paid enough attention to links between socio-economic conditions and other factors, including population composition, population density, the presence of migrant groups, and neighbourhood environmental conditions. This study examines DF and its distribution across 56 neighbourhoods of Jeddah City, Saudi Arabia, where the incidence of dengue remains high. Using stepwise multiple regression analysis it focuses on the key ecological correlates of DF from 2006-2009, the years of the initial outbreak. Neighbourhood variations in average case rates per 10,000 population (2006–2009) were largely predicted by the Saudi gender ratio and socio-economic status (SES), the respective beta coefficients being 0.56 and 0.32 (*p* < 0.001). Overall, 77.1% of cases occurred in the poorest neighbourhoods. SES effects, however, are complex and were partly mediated by neighbourhood population density and the presence of migrant groups. SES effects persisted after controls for both factors, suggesting the effect of other structural factors and reflecting a lack of DF awareness and the lack of vector control strategies in poorer neighbourhoods. Neighbourhood environmental conditions, as measured by the presence of surface water, were not significant. It is suggested that future research pay more attention to the different pathways that link neighbourhood social status to dengue and wider health outcomes.

## 1. Introduction

Since the 1980s the prevalence of dengue fever (DF) has increased rapidly and become problematic globally, but especially in low-and middle-income countries [1]. Like various strains of COVID, the spread of dengue has been associated with an increased globalisation of travel for work and leisure and increased urbanisation, which has created new environments of risk. Travellers may carry different variations of dengue serotypes and strains into areas where mosquitoes can spread the infection [2]. Rapid urbanisation also creates environmental conditions such as substandard housing, high population densities, poor sewer or waste management services and increased water insecurity, all factors which help create hospitable habitats for the *Aedes* mosquito. The combination of these factors creates optimal conditions for dengue transmission, and a considerable literature has evolved detailing the impact of different physical, demographic, and socio-economic environmental conditions associated with DF and its spread.

With respect to the physical environment, a common theme has been water insecurity. The shortage of water and unpredictability of supply in many areas, both urban and rural [3], deems it essential for inhabitants to keep water in large containers which, under the right temperature conditions, provide a suitable habitat for mosquitoes to breed [4,5]. For instance, found in Mexico and Nicaragua that, while the presence of a regular water supply was a protective factor against dengue, even people with a regular supply still stored water [6]. Similarly, in Brazil, found that households who had receptacles in the garden or courtyard or plants with temporary water pools on the property were more at risk [7]. The presence of containers as a mosquito habitat is not confined to non-western countries. It found also that a greater pupae density were in container habitats in lower socio-economic neighbourhoods in Baltimore and Washington DC, indicating the poorer quality of these environments [8].

Demographic factors, such as the density of a population in a community, may also be an important risk factor of dengue. A large number of studies, both urban and rural, link increased population densities to a higher prevalence of DF [3,9]. In cities such as Cali in Colombia, susceptibility to dengue was greatest in high density poorer neighbourhoods [10], while in Faisalabad, Pakistan, higher persons per room produced a similar result [11]. In Vietnam, a method called spatial analysis was applied to determine the densities of the human population (which was between 3000 to 7000 people per kilometre) which were susceptible to experiencing DF outbreaks [3]. There is also a possibility that the transmission of the dengue virus could appear in low density populations where a high mosquito to human ratio and lack of water exists [3]. In Thailand, for example, it was found that there were more cases of dengue fever in rural areas than in urban places; however, this does not indicate that urban centres do not significantly contribute to the spread of dengue [12]. The proportion of vector to host may be less suitable for serious transmission; nonetheless, the records of cases can still be considered as high. 

Migrant status has also been identified as a potential risk factor for the incidence and spread of dengue fever. Imported cases may arise because of the arrival of already infected migrant labour from countries with high rates of dengue [13,14,15], or from tourists returning from infected areas [16]. In a research review of migrant worker health in Singapore, argue that migrant workers appear to be at a higher risk of infectious diseases due to a complex interplay of factors; higher disease prevalence in their countries of origin, lower socio-economic status, including poorer living conditions, and problems of access to health services [17]. In a similar was found in Buenos Aires foreign workers tended to be clustered into low SES areas which were more likely to be subject to dengue fever outbreaks [14]. In the Middle East the impact of migrant populations on DF is likely to be magnified, particularly as they include religious pilgrims attending important events [18]. In Saudi Arabia, the number of dengue fever cases became increasingly concentrated among non-Saudi people, the ratio of non-Saudi to Saudi cases rising from 1.07 to 3.47 between 2011–2018 [19]. Compared to other environmental risk factors, the impact of migration on the incidence and spread of DF has been less well studied [19]. While migration may well be a risk factor for DF, its influence is highly dependent upon political and socio-economic conditions in migrants’ home countries [20], and the conditions they experience in places to which they move [21]. It is also dependent upon occupational type and the extent to which different types of jobs may differentially expose them to greater risks contracting dengue. Although not specifically focusing on dengue, argue that links between migration and health remain poorly understood and that a better understanding of such links remains a global public health priority [22].

Individual and neighbourhood socio-economic status will also affect the occurrence of dengue fever. A large number of studies in a variety of national contexts have shown that persons or areas of lower socio-economic status are related to an increased risk of dengue [11,23,24,25,26,27,28,29]. However, it has also been suggested that in certain contexts, more affluent neighbourhoods may also be at high risk. For example, in contrast to the findings in Cali, Colombia, where the incidence of DF exhibited a strong negative correlation to socio-economic status and relative population density [10], in Boa Vista, Brazil, found the reverse; the major risk areas were central high density neighbourhoods which contained the highest income population in the city [30]. In Jakarta, Indonesia, similarly concluded that high DF risk did not merely affect impoverished communities, but also wealthy populations [31]. The authors suggested that habits linked to rich populations, such as the growing of ornamental plants, the presence of swimming or ornamental pools in backyards or frequent travel to neighbouring endemic countries [16], may have contributed to this result. In the case of Jakarta, a contributing factor was that routine vector control was made more difficult in the wealthier neighbourhoods due to limited access given by the residents.

Such findings suggest that links between dengue incidence and socio-economic status are not straightforward and need to take account of other behavioural or environmental factors. An increasing number of studies thus have begun to take a broader perspective on the political ecology of mosquito habitats by examining sources of urban inequality and how they may contribute to the social distribution of dengue. Noteworthy here has been work which has examined the interrelatedness between the socio-economic environment and vector control strategies. For example, at the individual level, how higher SES relates to better DF knowledge and prevention practices has been an important line of enquiry [32,33,34,35]. However, less attention has been paid to the power dynamics in cities and how these have affected the social distribution of resources for effective for dengue control. Higher income neighbourhoods are also likely to have better water security [36], to live in higher quality housing [37], to live in lower density neighbourhoods [10], and to distance themselves from what they perceive to be other negative aspects of the urban environment which may put them at risk.

In light of the above discussion of the effects of different physical, demographic and social environments on DF risk, this study recognizes the significance of physical and social environmental factors in the neighbourhoods as reasons for the dispersion of dengue fever in Jeddah City, Saudi Arabia. Despite an increased incidence of DF in recent years [19], there have been few studies of the effect of environmental conditions on DF and especially of the relationship between socioeconomic and other environmental risk factors [18,38,39]. The large number of migrant workers and religious pilgrims, Saudi Arabia thus perhaps represents a unique case to examine relationships between DF risk and socio-economic, demographic and environmental factors [18].

This study poses three key questions relating to the distribution of DF in Jeddah City:

(i) To what extent is neighbourhood SES related to variations in DF numbers and case rates?
(ii) What other physical and social characteristics in neighbourhoods are most associated with DF case rates and how consistent is their impact through time?
(iii) What are the main paths between socioeconomic status and other environmental characteristics that affect DF case rates?

## 2. Methods

### 2.1. Study Area

The study area comprises Jeddah City, which is the second largest commercial centre in the Middle East, and with a population of 3.4 million (2010), is the fourth largest industrial city in Saudi Arabia. In that year half of Jeddah’s population were non-Saudi people, most of whom (64.6%), in contrast to the native Saudi population, were male. Migrants work in a range of industries including construction, as servants, and as car drivers, with most coming from Yemen, Egypt, and the Indian sub-continent. In addition to migrants seeking work, approximately 4–6 million people from all over the world travel to Makkah City, to the south of Jeddah, as Hajj pilgrims. The city has a very warm climate and this, combined with high relative humidity, has enhanced its vulnerability to dengue [40].

From 2005 onwards DF cases rose substantially and have remained high ever since [19]. In 2003 only 36 cases were recorded in Jeddah and 343 in Saudi Arabia as a whole, but by 2006 this had risen to 1307 and 1544, respectively, and to 2348 and 3302, respectively, by 2011. In the next six years case numbers fluctuated at high levels, reaching 4942 in Jeddah and 6345 in Saudi Arabia respectively by 2018. The paper focuses on the initial years of the outbreak (2006–2009) when detailed DF data was available at the neighbourhood level within Jeddah City. Traditionally the city has recorded more than three quarters of the reported DF cases in Saudi Arabia.

### 2.2. Data Sources

Four sources of data were used; (i) geocoded dengue fever data provided by the Health Ministry in Saudi Arabia for 2006–2009 enabled DF case numbers and rates to be calculated for neighbourhood areas; (ii) demographic data from the 2004 Census and neighbourhood population estimates for selected neighbourhoods from the Jeddah Urban Observatory for 2009. These two data sets enabled population estimates of neighbourhood populations to be made for each year, 2006–2009. In 2009 Jeddah City was divided into 112 neighbourhoods, but the absence of census data meant that the analysis was restricted to 56 neighbourhood areas for which demographic data was available. These neighbourhoods contained between 63.6% and 74.7% of all dengue cases in the city, 2006–2009 [41]; (iii) socio-economic status (SES). As no measures of socio-economic status are contained in the Saudi Arabian census, neighbourhood SES (high, medium and low) was estimated using the Delphi method [42], and the professional knowledge of 32 specialists in academia and financial institutions who worked in Jeddah City. More details are contained in Alkhaldy & Barnett [43]; (iv) Lastly, environmental factors relating to the presence of surface water, both within and adjacent to neighbourhood areas, were considered using data derived from Jeddah City records [44].

### 2.3. Variables

The dependent variable was the DF case rate per 10,000 population calculated for each neighbourhood area, for each year, and the average case rate for all years combined (2006–2009). The independent variables consisted of four measures relating to the socio-economic, demographic, migrant/cultural, and physical environment characteristics of the different neighbourhoods. All measures were interval scale variables, whether measured in terms of percentages or ratios. Socio-economic status, for example, was defined by the proportion of responses from key informants who categorised a neighbourhood as either high, middle, or low status. Demographic characteristics included three measures; population density (population per area in 2009), the presence of migrants (% population who were non-Saudi citizens in 2004) and the neighbourhood sex ratio (males per 100 females in 2004) which was determined for Saudi and non-Saudi residents. Because of the high dependence of Saudi Arabia on male migrant workers and because of institutional discrimination towards women, we speculated that male gender would also be an important risk factor for both non-Saudis and Saudis. Finally, two characteristics of the neighbourhood physical environment, relating to the presence of water hazards, were involved; the percentage of the total land area in a neighbourhood covered in surface water and the amount (measured in terms of km^2^) of surface water present within a two kilometre buffer area of neighbourhood centres. These data were only available for 2007–2009.

### 2.4. Research Method

To answer the first research question, a descriptive analysis was undertaken of DF case numbers and case rates to determine the degree to which they varied between high, middle and low SES neighbourhoods. The aim was to establish the extent to which there was a definite social gradient in cases.

With respect to the second question, the association between dengue fever case rates per 10,000 people in the period between 2006–2009 and all neighbourhood physical and social characteristics was originally calculated using bivariate correlations. This was followed by multivariate analyses using stepwise multiple regression, to estimate the relative explanatory power of the selected neighbourhood variables.

The third question sought to evaluate the value of different avenues which could possibly describe the relationship between socioeconomic status and rates of dengue cases. In this analysis we tested the significance of three selected pathways: (1) densities of neighbourhood population, (2) the existence of migrant groups, and (3) water hazards in the neighbourhood environment. We wished to establish the degree to which the correlation between neighbourhood socioeconomic status and DF case rates remained, after controlling for these possible intervening (confounding) factors. These analyses aimed to explore the different ways in which SES is connected to the DF case rates in Jeddah City neighbourhoods. We chose partial correlation, rather than mediation analysis, because the causal (mediating) links of the three possible intervening factors between neighbourhood SES and DF case rates remained unclear. We also tested for multicollinearity, but, despite some high intercorrelations between the Saudi and non-Saudi sex ratios, decided to include all variables in the model.

## 3. Results

### 3.1. Variations by Neighbourhood Socio-Economic Status

Table 1 indicates variations found in the number of dengue fever cases and the percentage per 10,000 people for the 56 neighbourhoods in Jeddah City grouped by neighbourhood socioeconomic status. Most neighbourhoods located south of Jeddah are of low socioeconomic status whereas high SES areas tended to be predominantly located in the middle and west of the city near the Red Sea. Middle SES neighbourhoods tended to be newer neighbourhoods located in the north and east of the central business district [43].

On average, low SES neighbourhoods contained the highest average of cases, which was 3.98 times more than high status neighbourhoods for the years 2006–2009. Overall, most (77.1%) dengue fever cases occurring in Jeddah City between 2006–2009 could be found in low SES communities. Middle and high status neighbourhoods had shares of 19.2% and 6.3% cases, respectively. However, neighbourhood social differences in case rates per 10,000 population were less marked. While low SES neighbourhoods still had the highest case rates, average rates in the most affluent neighbourhoods were also high. Nevertheless, the average case rate in low SES neighbourhoods (2006–2009) was still 1.41 times that of high SES areas.

**Figure 1:**
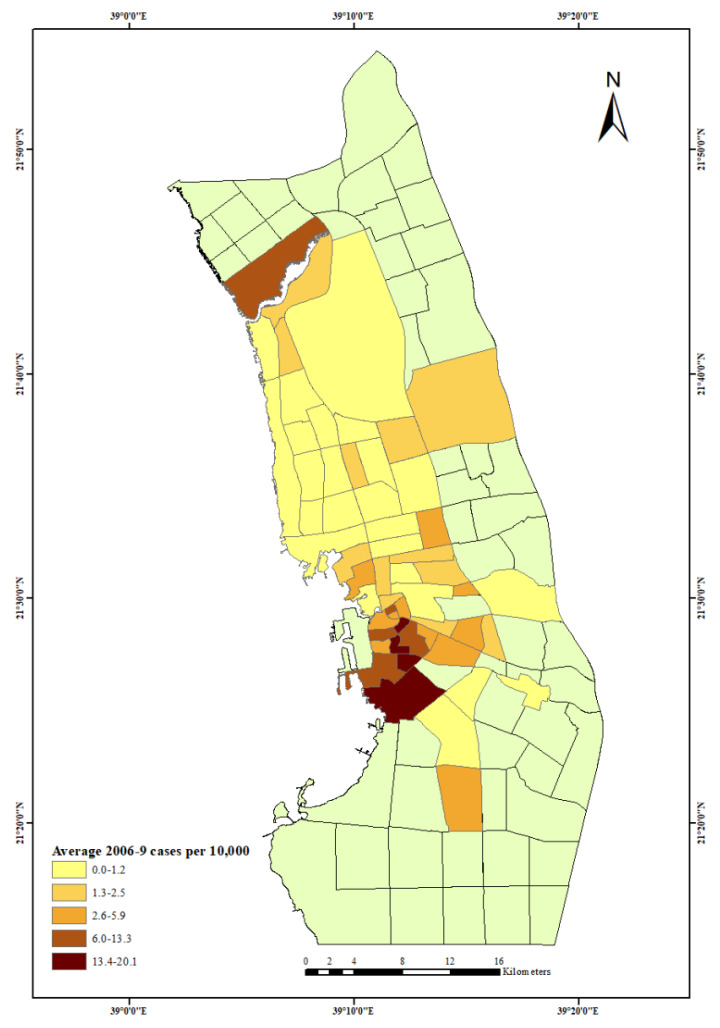
Average dengue fever cases per 10,000 people in Jeddah City, 2006–2009.

While low SES neighbourhoods saw a large number of cases and rates, substantial variation existed even among low status areas indicated by the high standard deviations. Some low SES areas had a noticeable increase in the number of cases while others had no change or a decrease. For example, in the inner city (Figure 1), two neighbourhoods, Al Azizeiah and Al Sabeel, immediately to the east and southeast of the port, had quite different trends; the Al Azizeiah neighbourhood recorded only one case in 2006, but 48 cases by 2009 and 94 overall. By comparison, the Al Sabeel neighbourhood had a similar number of cases (106) over the four year period, but these declined markedly from 71 cases in 2006 to only seven in 2009.

### 3.2. Neighbourhood Characteristics Impact on Dengue Fever Case Rates

#### 3.2.1. Bivariate Relationships

Table 2 illustrates the relationships between the case rates of dengue fever and the explanatory variables in the neighbourhoods of Jeddah City from 2006 to 2009. In 2006, neighbourhood socioeconomic status was only slightly related to the case rates that year. In 2007, this was not the issue when the Saudi and non-Saudi gender ratio and proportion of non-Saudi people were the only significant indicators. In 2008, a similar pattern was seen; however, at that time both population density in neighbourhoods and socioeconomic status were also substantial. The pattern in 2009 was different again with only the Saudi and non-Saudi gender ratios being important. Interestingly, in all the years mentioned, the existence of surface water had no relationship to DF case rates. Thus, while neighbourhood SES status was the only variable that was initially significant, the results for the average case rate 2006–2009 suggest that it can act independently or together with other factors. Furthermore, two other significant variables which did not change were the Saudi and non-Saudi gender ratios. With the exception of 2006, these factors had a continuous effect on DF case rates.

#### 3.2.2. Multiple Regression Models for Case Rates of Dengue Fever

The model of multiple regression was applied to examine the connection between the dengue fever case rate and social and physical neighbourhood elements in Jeddah City neighbourhoods. The modelling was conducted in three steps. The first step involved examining the relationship between neighbourhood gender ratios (for both Saudi and non-Saudis) and the presence of migrant groups (percent of non-Saudi) and DF case rates. Next, neighbourhood SES and population density variables were entered into the model. Lastly, the neighbourhoods’ physical attributes, as shown by the existence of surface water, were incorporated.

Stepwise multiple regression models for dengue fever case rates in Jeddah City neighbourhoods from 2006 to 2009 are shown in Table 3. For neighbourhood population composition, with the exception of 2006, the Saudi sex ratio (MFS) was significant in all years, showing that Saudi males experienced more dengue cases than Saudi females. By comparison, the non-Saudi gender ratio (MFNS) was no longer substantial once the Saudi gender ratio (MFS) was inputted into the model, mainly because MFS and MFNS had a strong correlation. The second model shows how incorporating the percentage of low SES (LSES) and population density (PopDen) impacted the results. Though the Saudi gender ratio continued to be the main predictor of dengue fever case rates in three of the four years, neighbourhood socioeconomic status was still considered to be significant in 2006, 2008 and in all years together (2006–2009). Population density in 2007 displayed an independent effect, while the proportion of non-Saudi people (NS) remained important only in 2008 once socioeconomic status was taken into account. The environmental risk factor, indicated by the presence of surface water, both within and adjacent to neighbourhood areas, was not significant in any year.

### 3.3. Pathways between Neighbourhood Socioeconomic Status and Other Explanatory Factors

Whereas neighbourhood male to female ratios seems to be the most consistent factor affecting dengue’s distribution, it is clear that socioeconomic status of neighbourhoods has also had an instrumental role, especially since the majority of dengue fever cases (77.1%) appeared in neighbourhoods of low socioeconomic status. Given that low SES neighbourhoods were mostly areas with high densities of population (r = 0.59; *p* < 0.00) and were comprised of large non-Saudi migrant populations (r = 0.50; *p* < 0.00), in this last section we explore some of the relationships between these features.

Table 4 shows partial correlations between dengue fever rates and socioeconomic status in neighbourhoods after creating controls, first for density of population, and second for density population and the percentage of the population that was non-Saudi. Neither population density nor the proportion of non-Saudi indicates any connection to dengue fever case rates in 2006; therefore, it is not surprising that controls for both variables had little effect on the relationship (r = 0.30) between dengue fever case rates and neighbourhood socioeconomic status. During a time in 2007, when cases were very few, there was no apparent relationship between dengue fever case rates and low socioeconomic status. Mechanisms for population density (not significant) and non-Saudi (r = 0.36; *p* < 0.00) had no effect on this situation.

Low socioeconomic status (r = 0.48), population density (r = 0.31) and non-Saudi people (r = 0.53) in 2008 were all factors that significantly affected the dengue fever case rate. Controls for the density of population decreased the dengue-neighbourhood SES relationship to some degree by 0.38 (*p* < 0.00) and controls for both population density and the proportion of non-Saudi people to 0.25 (*p* < 0.06), indicating that both factors facilitated the relationship between the dengue fever case rate and neighbourhood socioeconomic status, but not completely.

Again in 2009, low socioeconomic status displayed no significant connection to dengue fever case rates (r = 0.19; however, it did so once population density (partial r = 0.32; *p* < 0.02) was controlled and slightly when both population density and non-Saudi population were taken into consideration (partial r = 0.24; *p* < 0.08). In the combined years of 2006–2009, a clearer situation was more obvious when low socioeconomic status (r = 0.36) and non-Saudi people (r = 0.34), but not the density of population, were greatly linked to dengue fever case rates. Controls for both population density and non-Saudi people reduced but did not completely exclude the correlation (partial r = 0.31; *p* < 0.02) between the % of low SES and dengue fever case rates, signifying possibly the presence of a lifestyle pathway connecting low socioeconomic status and dengue fever cases.

These combined results imply that population density and the presence of non-Saudis may perform together to influence the number of dengue fever case rates. The effect of having non-Saudis living in the area was more stable than density of population indicating that neighbourhoods with high population density are more likely to have a higher rate of dengue fever cases when non-Saudi migrant populations reside there. Controlling both variables greatly reduced the connection between low socioeconomic status and dengue fever case rates, especially in 2008, and for the whole period of 2006–2009. It was not apparent in 2009 when controls showed a much stronger connection between dengue fever case rates and low socioeconomic status.

While the effects of population density and the presence of migrant groups may partly account for the relationship between SES and dengue case rates, the above analysis ignores the effects of gender differences that were so evident in Table 3. With this in mind, Table 5 shows gender differences in the overall number of DF cases and case rates stratified by neighbourhood SES for Saudi and non-Saudi populations. It shows, for both Saudis and non-Saudis, that gender differences in DF case numbers and case rates were greatest in less wealthy neighbourhoods. This distinction was most noticeable for non-Saudis but still evident for the Saudi population. While both groups demonstrated a social gradient in the number of cases and case rates, the proportion of cases occurring in low SES neighbourhoods was greater for non-Saudis (81.2%) than for Saudis (72.0%), reflecting the concentration of migrants in poorer parts of the city. It is interesting that in the poorest neighbourhoods, while case rates were similar for Saudi (18.3) and non-Saudi (18.8) males, this was not true for females, with Saudi females having higher rates than their non-Saudi counterparts (12.3 versus 8.1). Although more research is needed on this issue, it is likely that this reflects the increased reliance on migrant females for domestic work, as Saudi females have increasingly entered the formal workforce and been subject to higher levels of DF exposure [21]. It could also reflect the increased presence of non-Saudi females working in the safer environments provided by higher and middle income Saudi households.

Table 2 and Table 3 revealed that having surface water in a neighbourhood and its surroundings was not considered as being important in determining variations in DF rates in Jeddah City neighbourhoods. It was also found that there was no significant relationship between the existence of surface water and socio-economic factors (Table 6). Only in 2008 did the correlation between the neighbourhood SES and the presence of surface water within the 2 km buffer area approach significance (r = 0.23; *p* < 0.09). While this suggested that poorer neighbourhoods had more exposure to surface water within a two-kilometre buffer zone, the fairly large standard deviations suggest that substantial differences in exposure to the existence of surface water were present in low status Jeddah City neighbourhoods.

## 4. Discussion

This study aimed to explore various reasons for neighbourhood differences in dengue fever in Jeddah City neighbourhoods during the early years of the initial outbreak (2006–2009). Five key findings emerged.

First, the pattern of neighbourhood variations changed rapidly over this period, reflecting the quick spread of the virus into different types of neighbourhoods. Low socioeconomic status appeared to be the single important variable in 2006. However, in following years, more factors appeared, and for 2006–2009 as a whole, DF rates were highest in low SES neighbourhoods with high proportions of Saudi and non-Saudi males, and in those which contained a higher proportion of non-Saudis. Multivariate analysis suggested that neighbourhood male/female sex rations for both Saudis and non-Saudis had the most consistent effect on case rates. This indicates that the manner in which dengue fever case rates appeared has become more complicated with the male to female balance and SES of neighbourhoods interacting in subtle ways with the presence of migrants and population density. Although the time period of the study was not long enough, it is likely that neighbourhood social differences, while marked at the start of the epidemic, became less marked as the virus spread to other neighbourhoods. With public health measures, neighbourhood social differences are likely to intensify again as these are most likely to be followed up in higher status neighbourhoods.

A second important finding was the persistent link between the gender balance of a neighbourhood and case rates. One possible explanation for the high positive correlation between neighbourhood sex ratios may be related to the culture in Jeddah. It appears that culture is quite influential because Saudi women, reflecting established gender discrimination, are mainly forced to stay at home or near their home. Until recently, females were not permitted to drive a car or to work far from their home, and consequently there were not many work opportunities for women [45]. Because of this, Saudi (and non-Saudi) males tend to live more active lifestyles around the city, and because they are not at home much of the time, they risk contracting dengue fever [46]. However, it is interesting that gender differences were less for Saudis than non-Saudis, with Saudi females having higher case rates than non-Saudi females, especially in poorer neighbourhoods. This finding deserves further examination but could reflect the fact that non-Saudi females may act as servants in middle and higher income neighbourhoods and thus receive a greater level of protection than their Saudi female counterparts.

A third important finding is that while differences among males and females between neighbourhoods were important determinants for getting dengue, the influence of neighbourhood socioeconomic status was also great, as it was a statistically significant forecaster of the dengue fever case rate in 2006 and 2008 and overall for the years 2006–2009. The Saudi pattern thus conforms to many other studies which have indicated that DF case rates are highest in low SES neighbourhoods. However, the pathways linking SES and DF outcomes are complex. The partial correlation analysis suggested that two main pathways link neighbourhood SES to DF case rates. First, it found in Cali, Colombia, neighbourhood SES was highly related to neighbourhood population density [10]. Second, and similar to the findings was in Buenos Aires, Argentina, foreign migrant groups tended to be clustered in low SES areas [14]. People of non-Saudi descent living in low socioeconomic neighbourhoods in Jeddah City experience the poorest housing and environmental circumstances. Many of the old homes had no air conditioning, and people hardly ever closed their windows allowing for the *Ae. aegypti* mosquitoes to easily come into the home. The worst case rates were for foreign males living in the poorest areas. While the analysis found evidence for both pathways, these were not a full explanation of the high rates of dengue in poor neighbourhoods.

A fourth important finding was thus that controls for density of population and migrant status reduced, but did not eliminate, the connection between neighbourhood socioeconomic status and dengue case rates. This implies that a third pathway may be possible, reflecting the impact of cultural factors, in particular resistance to dengue control efforts. International research suggests that persons with a low level of education are likely not to fully comprehend the risks of contracting dengue [33,35], and may also both be more distrustful of government vector control efforts. For example, in Saudi Arabia, culture has a restrictive effect on spray control of *Ae. aegypti* in the home. In Saudi culture, it is typical for only men to respond to and open the door for public health workers. Therefore, it is difficult to connect with low-income groups and inform them about the necessity to control dengue and be able to recognise its symptoms. Displaying resistance to public health programmes has been quite the norm in other countries, so this could possibly explain the variations in neighbourhoods and the occurrence of dengue fever cases in Jeddah City. However, the fact that the average dengue case rates for 2006–2009 were also relatively high in the most affluent neighbourhoods deserves further attention. A similar pattern also found in Boa Vista, a regional capital in Brazil [30], as did in Indonesia [31]. In the latter case, routine vector control and monitoring could not be effectively implemented in the neighbourhood due to limited access given by the residents.

A final important finding was that, in contrast to much other research, environmental issues relating to the presence of surface water were not significant predictors of dengue case rates in Jeddah City. Furthermore, there was no significant relationship between neighbourhood socioeconomic status and the presence of surface water. Despite there being some indication that poorer neighbourhoods had more exposure to surface water within the two-kilometre buffer zone, the fairly large standard deviations show substantial differences in the amount of water that was actually there within Jeddah City neighbourhoods. One reason why there was no direct relationship between the amount of surface water and rates of dengue fever in Jeddah City is mainly because after 2007, when the government control strategies dealt with the problem of dengue fever and surface water, they were considered effectively under control. These strategies were successful due to the workers being very conscientious in their endeavours to fix the situation. Since much of the surface water could be seen, any changes could simply be observed.

However, the extent to which water security issues remain important is unclear. Unfortunately, Jeddah City has had many problems in handling their water issues due to having a shortage of water and a lack of water resources. Consequently, residents in poor neighbourhoods frequently store their water in tanks and, because most of the tanks are in poor condition, water frequently leaks out of them. Because Jeddah City has an inadequate sewerage network, sewage can be found leaking into the groundwater, which contributes to a problem that already exists. Thus, in this respect, links between the quality of urban physical and social environments suggest that mosquito habitats in Jeddah seem similar to that of many other cities in the non-western world [36].

## 5. Conclusions

In examining the situation in Jeddah, the city seems to have many factors influencing its high rates of dengue fever, together with warm climatic conditions, rapid growth in urban areas, the high number of the non-Saudi population, and migration. Additionally, aspects of the urban ecology such as high housing densities, especially in poorer neighbourhoods, have enhanced neighbourhood inequalities in exposure to risk factors and dengue outcomes.

While Jeddah City shares many common characteristics with cities elsewhere in the non-western world, the findings of our study cannot easily be generalised to other non-western countries experiencing a surge in dengue cases. While there are some common themes, such as the relationship between SES and DF case rates, other aspects of the Jeddah City case are relatively unique. Particularly significant here is the very high dependence of the city on migrant labour to fulfil its employment needs. During the initial dengue outbreak, poorer and particularly non-Saudi neighbourhoods were most at risk of dengue which, despite the implementation of control strategies beginning in 2007, currently remain at high levels. Furthermore, greater understanding is needed with regard to the implementation of DF containment policies and how, and where, these have been implemented and their effect on the social distribution of dengue. 

Given this situation, a number of questions present themselves. First, to what extent have neighbourhood inequalities in the risk of exposure intensified and to what extent have control strategies targeted poorer neighbourhoods? While the policy to eradicate standing water bodies appears to have had an impact, it remains unclear regarding the extent to which water security issues remain important, especially in poorer neighbourhoods with intermittent supplies of water. Also unclear is the extent to which vector control strategies have been ineffective when faced with cultural barriers, especially in low income migrant neighbourhoods. This is a critical issue, especially since differences in the number of reported cases, while declining between 2011–2018 for the Saudi population, have rapidly increased for non-Saudis [19]. Finally, greater understanding of the links between the types of migration and dengue is needed for both more permanent work migrants and religious pilgrims in order to develop more evidence-based guidelines for dengue and other disease control strategies [47].

This research has provided an overview of some of the factors important in the initial stages of the early dengue outbreak, but has many limitations. These include the very poor quality of Saudi census data and the lack of information on dengue cases at the neighbourhood level since 2010. In addition, the absence of any SES measure required the construction of a surrogate measure based on informant interviews of key contacts in Jeddah. While this analysis was able to measure socio-economic trends in the incidence of dengue between 2006–2009, it did not examine the detailed aspects of the local urban ecology that have aided the spread of dengue across the city since then.

It is important, therefore, that key government agencies in Saudi Arabia and Jeddah pay attention to the importance of local neighbourhood factors in their formulation and implementation of dengue control policies. Saudi Arabia is a wealthy country, yet its success in limiting the rise in dengue fever cases has been poor. Only when better information systems and more effective implementation strategies are developed will dengue perhaps be significantly reduced in hotspot cities like Jeddah, and perhaps in Saudi Arabia as a whole. 

## Figures and Tables

**Table 1:** Average number of dengue fever cases and case rate per 10,000 people by neighbourhood socioeconomic status, Jeddah City, 2006–2009.

	Mean			Standard Deviation		
Year	High SES	Mid SES	Low SES	High SES	Mid SES	Low SES
Cases 2006	11.2	7.0	27.2	16.8	7.3	30.8
Cases 2007	1.1	2.0	4.0	0.8	3.1	4.5
Cases 2008	1.6	5.8	17.8	1.4	7.3	16.4
Cases 2009	5.4	11.7	27.3	5.9	15.1	23.8
Average cases 2006–2009	4.8	6.6	19.1	5.2	6.9	15.3
Rate 2006	6.2	2.4	6.5	11.5	3.2	7.5
Rate 2007	0.7	0.3	1.2	0.9	0.4	2.4
Rate 2008	0.5	1.9	4.7	0.4	3.6	5.3
Rate 2009	6.0	3.1	6.7	8.3	7.1	9.2
Average rate 2006–2009	3.4	1.9	4.8	4.7	3.9	4.5

**Table 2:** Correlations between dengue fever case rate and the explanatory variables in Jeddah City neighbourhoods, 2006–2009.

Explanatory Variables	Rate Cases, 2006		Rate Cases, 2007		Rate Cases, 2008		Rate Cases, 2009		Rate Cases, 2006-2009	
	Cor	*p*<	Cor	*p*<	Cor	*p*<	Cor	*p*<	Cor	*p*<
PopDen	0.12	0.37	0.02	0.89	0.31	0.02	−0.12	0.37	0.07	0.58
MFS	−0.09	0.52	0.93	0.00	0.64	0.00	0.68	0.00	0.58	0.00
MFNS	−0.08	0.54	0.93	0.00	0.63	0.00	0.68	0.00	0.58	0.00
NS	0.09	0.50	0.36	0.00	0.53	0.00	0.23	0.08	0.34	0.01
LSES	0.30	0.02	0.15	0.28	0.48	0.00	0.19	0.17	0.36	0.00
Water7	-	-	0.08	0.53	-	-	-	-	-	-
Water8	-	-	-	-	0.06	0.65	-	-	-	-
Water9	-	-	-	-	-	-	0.09	0.50	-	-
WaterK07	-	-	0.08	0.55	-	-	-	-	-	-
WaterK08	-	-	-	-	0.14	0.30	-	-	-	-
WaterK09	-	-	-	-	-	-	0.16	0.24	-	-

PopDen: Population density (2009), MFS: sex ratio Saudi males per 100 females, 2004; MFNS: sex ratio non-Saudi males per 100 females, 2004; LSES: percent of respondents who ranked the neighbourhood as one of low socio-economic status (2013); Water7 to Water9: surface water percentage of neighbourhood land area (2007–2009); WaterK07 to WaterK09: surface water in areas surrounding the neighbourhood within a 2km radius (2007–2009).

**Table 3:** Multiple regression using population and socioeconomic explanatory variables, 2006–2009.

Explanatory Variables	Step Entered at	Predictor Variables	Adjusted R^2^	Beta Coefficient	*p*-Value
Population composition only					
Rate Cases, 2006	-	-	-	-	-
Rate Cases, 2007	1	MFS	0.86	0.93	0.00
	2	MFS and NS	0.88	0.90/0.12	0.00
Rate Cases 2008	1	MFS	0.39	0.64	0.00
	2	MFS and NS	0.53	0.54/0.39	0.00
Rate Cases, 2009	1	MFS	0.45	0.68	0.00
Rate Ave Cases 2006–2009	1	MFS	0.33	0.58	0.00
All variables					
Rate Cases, 2006	1	LSES	0.07	0.30	0.00
Rate Cases, 2007	1	MFS	0.86	0.93	0.00
	2	MFS and PopDen	0.89	0.95/0.15	0.00
Rate Cases, 2008	1	MFS	0.39	0.64	0.00
	2	MFS and LSES	0.57	0.60/0.43	0.00
	3	MFS, LSES, NS	0.61	0.55/0.32/0.23	0.00
Rate Cases, 2009	1	MFS	0.45	0.68	0.00
RateAve Cases, 2006–2009	1	MFS	0.33	0.58	0.00
	2	MFS and LSES	0.42	0.56/0.32	0.00

**Table 4:** Partial correlations between dengue fever case rates and neighbourhood socioeconomic status controlling for different explanatory factors.

Variables	Control Variables *	Partial Correlations	*p*-Value
Rate Cases, 2006 and LSES **	PopD09	0.28	0.04
	PopD09 and NS	0.28	0.04
Rate Cases, 2007 and LSES	PopD09	0.17	0.22
	PopD09 and NS	0.03	0.82
Rate Cases, 2008 and LSES	PopD09	0.38	0.00
	PopD09 and NS	0.25	0.06
Rate Cases, 2009 and LSES	PopD09	0.32	0.02
	PopD09 and NS	0.24	0.08
Rate Cases, Ave 2006–2009 and LSES	PopD09	0.40	0.00
	PopD09 and NS	0.31	0.02

* Excluded surface water characteristics as no significant correlations existed with neighbourhood SES. ** LSES: neighbourhood scores based on % ranks by Delphi respondents indicating low SES status.

**Table 5:** Number of dengue fever cases and case rates per 10,000 by gender and migrant status by neighbourhood socioeconomic status, 2006–2009.

Neighbourhood SES		Saudi			Non-Saudi		
		Male	Female	Difference	Male	Female	Difference
High	Number of cases	45	24	21	32	8	24
	Rate cases per 10,000	5.8	3.2	2.6	4.9	2.2	2.6
Middle	Number of cases	175	100	75	212	36	176
	Rate cases per 10,000	7.4	4.5	2.9	9.9	2.7	7.0
Low	Number of cases	549	337	212	1002	238	764
	Rate cases per 10,000	18.3	12.3	6.0	18.8	8.1	10.7

**Table 6:** Percentage of neighbourhood land area covered in surface water and surface water area within a two kilometre buffer area (km^2^).

Surface Water Percentage								
Area SES	2007		2008		2009		Change 2007–2009	
	Mean	SD	Mean	SD	Mean	SD	Mean	SD
High	0.30	0.66	0.09	0.23	0.04	0.10	−0.26	−0.57
Med	0.16	0.24	0.04	0.08	0.32	1.01	0.17	0.77
Low	0.31	0.58	0.10	0.19	0.15	0.51	−0.16	-0.06
Surface water in two kilometre buffer area size (km^2^)								
High	8.43	12.21	6.35	12.67	3.60	6.00	−4.83	−6.21
Med	11.67	14.36	7.55	11.45	9.30	16.54	−2.37	2.18
Low	14.09	18.15	12.09	18.91	24.30	94.74	10.21	76.58

## Data Availability

The data presented in this study are available on request from the corresponding author.
